# Strain evaluation of axially loaded collateral ligaments: a comparison of digital image correlation and strain gauges

**DOI:** 10.1186/s12938-023-01077-z

**Published:** 2023-02-11

**Authors:** Gwendolin Prusa, Leandra Bauer, Inês Santos, Christoph Thorwächter, Matthias Woiczinski, Manuel Kistler

**Affiliations:** Department of Orthopaedics and Trauma Surgery, Musculoskeletal University Center Munich (MUM), University Hospital, LMU Munich, Marchioninistraße 15, 81377 Munich, Germany

**Keywords:** Cadaveric biomechanical study, Collateral ligament, Digital image correlation, Strain analysis, Strain gauge

## Abstract

The response of soft tissue to loading can be obtained by strain assessment. Typically, strain can be measured using electrical resistance with strain gauges (SG), or optical sensors based on the digital image correlation (DIC), among others. These sensor systems are already established in other areas of technology. However, sensors have a limited range of applications in medical technology due to various challenges in handling human soft materials. The aim of this study was to compare directly attached foil-type SG and 3D-DIC to determine the strain of axially loaded human ligament structures. Therefore, the medial (MCL) and lateral (LCL) collateral ligaments of 18 human knee joints underwent cyclic displacement-controlled loading at a rate of 20 mm/min in two test trials. In the first trial, strain was recorded with the 3D-DIC system and the reference strain of the testing machine. In the second trial, strain was additionally measured with a directly attached SG. The results of the strain measurement with the 3D-DIC system did not differ significantly from the reference strain in the first trial. The strains assessed in the second trial between reference and SG, as well as between reference and 3D-DIC showed significant differences. This suggests that using an optical system based on the DIC with a given unrestricted view is an effective method to measure the superficial strain of human ligaments. In contrast, directly attached SGs provide only qualitative comparable results. Therefore, their scope on human ligaments is limited to the evaluation of changes under different conditions.

## Background

Functions of the human body ligaments include transmitting tensile forces to surrounding structures and limiting the range of motion to a physiological extent. The response of soft tissues, such as ligaments to an applied load or force can be determined by strain measurement, which is a key parameter during experimental testing of joint kinematics using actively controlled motion test rigs. Generally, strain is defined as the relative change in length under load divided by the initial length [15, 42]. Between 2 and 4% strain, ligaments show an elastic behavior, which allows the measurement of mechanical properties according to the Hooke’s law [1, 27, 34]. However, the characterization of the mechanical properties of ligaments is challenging due to its inhomogeneous nature, shape, or position. In addition, it might be necessary to measure strains in both optically accessible and inaccessible regions. Currently, there is no standardized method to assess in vitro strain, which limits comparability between different publications. Several sensors with different functionalities are available to the engineering field. A general distinction is made between contact and noncontact measurement methods. Contact measurement techniques include, for example, directly attached strain gauges (SG) or differential variable reluctance transducers (DVRTs). DVRTs measure displacement by the movement of a magnetic core within two coils of wire inside of the sensor. Previous studies used DVRTs to determine strain of the anteromedial bundle of anterior cruciate ligament (ACL) [8, 43] or the superficial medical collateral ligament (MCL) [32] within various loading conditions. Although the strain measurement using DVRTs is highly accurate, it also has its downsides like the damage of underlying tissue due to the fixation using barbed screws or the importance of free access through dissection to avoid the interference with other structures. In contrast, foil-type SGs are less expensive and destructive as compared to DVRTs, being a gold standard for the strain measurement on bone, but rarely used on soft tissue [4, 9, 28, 35]. The electrical resistance of the measuring grid within the SG changes when a mechanical load is applied, which requires a lossless transmission of strain between SG and the measured object [15]. Prior studies from Clark et al. [4] and Norton-Old et al. [28] used cyanoacrylate adhesives for a lossless transmission between foil-type SG and human soft tissues because of its almost instantaneous fixation [4, 35]. These investigations have shown that using SGs promises repeatable measurements but only a qualitative representation of the process up to an elongation of 5% [15].

Noncontact measurement methods, like optical sensor devices based on the digital image correlation (DIC) or ultrasound (US), do not directly interfere with the specimen. DIC, which assesses the full-field (3D) surface strain of an object, is based on the detection of temporal change of a stochastic pattern on the surface of a measurement object due to an external load [38]. 3D-DIC is now widely used for soft tissue biomechanical testing like tensile tests, but obtaining random patterns with optical contrast on the tissue surface can be challenging [31]. Most commonly, black-on-white patterns are used for light-colored objects, such as bones, but white-on-black speckle patterns are better suited for slightly darker ligaments [20, 22, 31]. Previous studies compared the strain values of the DIC method with different ultrasound approaches [5, 12, 29]. These studies used different testing parameters and ligaments like axial loading of the lateral collateral ligament (LCL) to 5% strain [12], cycled loading of a rat’s Achilles tendon between 0 and 1% strain [29] or varus/valgus loading of the medial and lateral collateral ligament with a total knee implant [5]. Although US-based strain measurement might be used in as in vivo application, it is limited to 2D deformations and highly depending on the image quality. Nonetheless, these studies validated the use of the 3D-DIC method as a highly accurate and reproducible tool for strain measurement on soft tissue.

In summary, unlike all other systems and despite being a promising method to measure strain (especially in optically inaccessible regions), the assessment of ligament strain with directly attached foil-SG has not yet been established. Therefore, the main objective of this study was to compare and evaluate the strain of human collateral ligaments under uniaxial cyclic loading using SG and 3D-DIC. We hypothesized that strain can be measured with both 3D-DIC and foil-type SG on human soft tissue. Furthermore, it is assumed that foil-type SGs are also a valid method to measure local longitudinal strains directly on ligaments and consequently their results are comparable to established systems such as 3D-DIC.

## Method

### Specimen preparation

A sample of 18 fresh frozen knee joints with a mean age of 51.9 ± 17.4 years were used in this study. The sample size was pragmatically chosen to establish a valid experimental method. The specimens had been used in a previous test for which they were shortened 20 cm proximal and 22 cm distal to the epicondylar line and stored at − 20 °C after the experiments [2, 6]. During this previous testing process, the ligaments did not undergo any elongation and were not visibly damaged. For the present study, each specimen was allowed to thaw 24 h prior to dissection at room temperature. All unnecessary tissue was removed and only the lateral and superficial medial collateral ligaments with their bony attachments were preserved. During the dissection, the length of the MCL and LCL was recorded at full extension using a calliper [30]. Their attachment sites at the femur, fibula and tibia were identified according to those described in literature [18, 19].

**Fig. 1:**
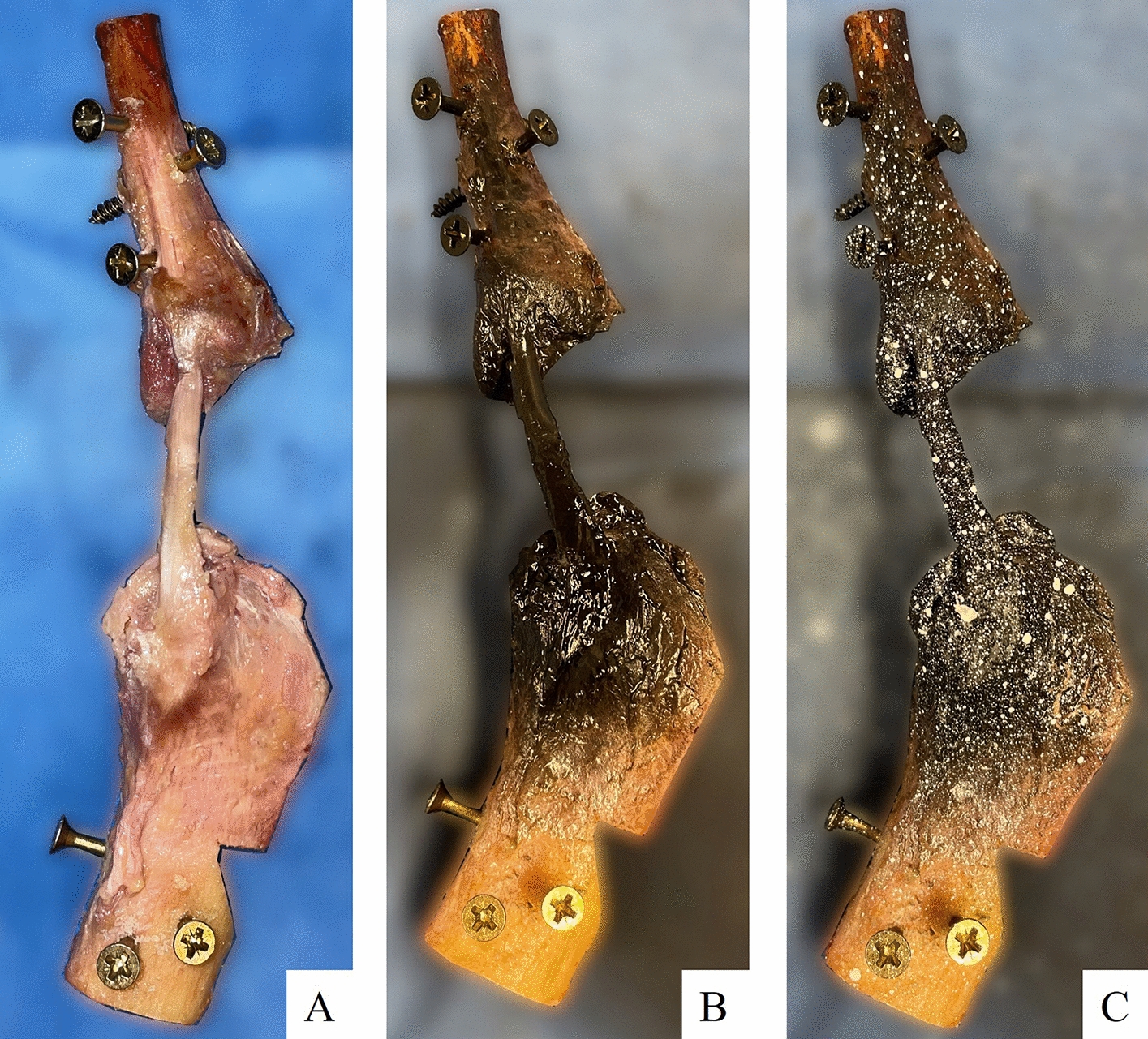
Preparation of the bone−LCL−bone complex (**A**) with black coat (**B**) and white speckle pattern (**C**) for the 3D-DIC measurement

For the individual testing of the ligaments a bone−ligament−bone unit was generated. Therefore, a saw was used to section the femoral and tibial segments in the sagittal plane at the intercondylar notch, and the tibia and fibula were separated by dissection of the tibiofibular joint. Throughout the preparation, the specimens were moistened with saline solution. For better fixation of the bony parts in the experimental setup, wedges were cut into the bones and screws were attached (Fig. 1A). After preparation, the bone−ligament−bone units were stored again at − 20 °C until one day before testing. The surfaces of the ligaments and SG were prepared for the 3D-DIC measurement by applying a thin coat of black spray paint (Deco Matt, European Aerosols GmbH, Hassmersheim, Germany) and a speckle pattern using white spray paint (Aqua Eco + , European Aerosols GmbH, Hassmersheim Germany) (Fig. 1B, C).

### Material testing

**Fig. 2:**
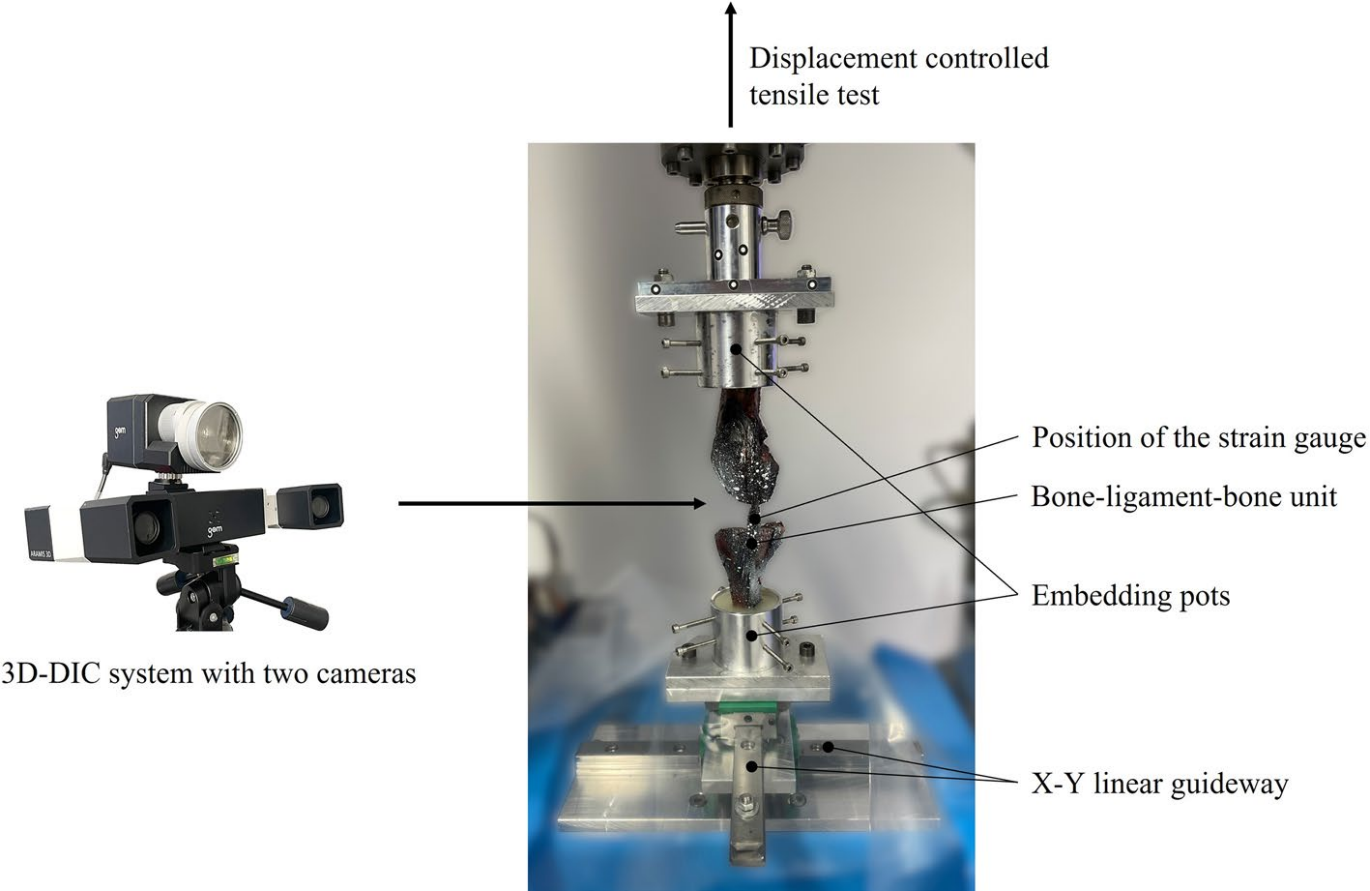
Custom-made setup for the testing of bone−ligament−bone complex and strain assessment using 3D-DIC and SG

The bone−ligament−bone complexes were subsequently prepared by embedding the femoral end in an aluminum pot using screws and resin (RenCast^®^ FC 52/53 Isocyanate & FC 53 Polyol, Huntsman Advanced Materials (Europe) BV, Everberg, Belgium). Afterwards, the aluminum pot was fixed to the upper part of the testing rig and the force of the material testing machine (Zwick Z10, Zwick GmbH & Co. KG, Ulm, Germany) was zeroed to track the force effect of curing. Subsequently, the tibial/fibular end of the ligament was embedded to the lower part of the rig in another aluminum pot. This lower pot was fixed on an X–Y linear guideway (Schaeffler Technologies AG & Co. KG, Herzogenaurach, Germany) to ensure alignment of the longitudinal fibers and to avoid shear load (Fig. 2).

For all measurements the engineering strain was assessed experimentally, (a) using the elongation of the testing machine as a reference (REF) and (b) by the 3D-DIC system. The 3D-DIC system with a resolution of 1936 × 1216 pixels (ARAMIS 3D Camera 2.3 M, GOM GmbH, Braunschweig, Germany) was positioned in front of the testing rig. A measuring distance of around 700 mm was adjusted using the linear positioning stage of the system to match the center of depth of the measuring volume (140 mm × 90 mm × 90 mm). Within the measuring plane the approximate measuring accuracy is 2.8 µm in-plane and 5.6 µm out-of-plane, and the accuracy of strain measurements are approximately 0.1%. After the specimen was positioned in the testing rig, a reference image was captured from the unloaded condition of the specimen before each test. During the test trials, the images were acquired at five frames per second (5 Hz). For the comparison of the strain assessment techniques (3D-DIC, REF, and SG) the bone−ligament−bone unit was always preloaded up to 2 N to straighten the fibers. After that 15 loading cycles at a rate of 20 mm/min up to an elongation of 2 mm (displacement control) were applied. A total of two test trials were performed under these conditions with a resting period of at least 10 min in between (Table 1). The testing parameters were chosen based on the previously published studies to minimize the load hysteresis of the ligament [33, 37, 40, 44, 47].

**Table 1:** Description of the different test trials

Trial 1	Trial 2
• Preload: 2 N • Cyclically loaded (displacement control) • Strain measurement: REF, 3D-DIC	• Preload: 2 N • Cyclically loaded (displacement control) • Strain measurement: REF, 3D-DIC, SG

In the second trial, in addition to the 3D-DIC system and REF, strain was recorded with a directly attached SG. For this purpose, a linear foil-SG (1-LCY41-3/350ZE, Hottinger Brüel & Kjaer GmbH, Darmstadt, Deutschland), was used. Before testing, a speckle pattern was applied on the surface of the SG to determine the influence of the SG obtained by 3D-DIC. To achieve a lossless transmission of strain between SG and ligament, a drop of superglue based on Cyanacrylat was applied on the back of the SG and attached to the center of the surface of the ligament, which was estimated using a ruler, in direction of the fibers [4, 21]. The shunt calibration and data acquisition of the SG were controlled by a custom-made LabVIEW (Version SP1 2014, National Instruments, Austin, TX, USA) program, using a compactDAQ system (NI cDAQ 9174, National Instruments) and a hardware module (NI9236). To allow synchronization of the sensor systems, a second hardware module (NI9402) for measurement of a digital TTL (transistor−transistor−logic) trigger signal from the 3D-DIC system was implemented.

### Data analysis

To compare both strain assessment techniques, a particular reference strain was required. The reference strain (ɛ_REF_), which was measured though the elongation of the displacement controlled experimental setup, was described by the difference of the current position of the material testing machine (*l*), its first position (*l*_0_), as well as the initial length of the ligament (*l*_Lig_). According to these parameters, ɛ_Ref_ was calculated as follows:

1 $${\varepsilon }_{\mathrm{REF}}= \frac{l- {l}_{0}}{{l}_{\mathrm{Lig}}} \cdot {10}^{2} \left[\%\right].$$

**Fig. 3:**
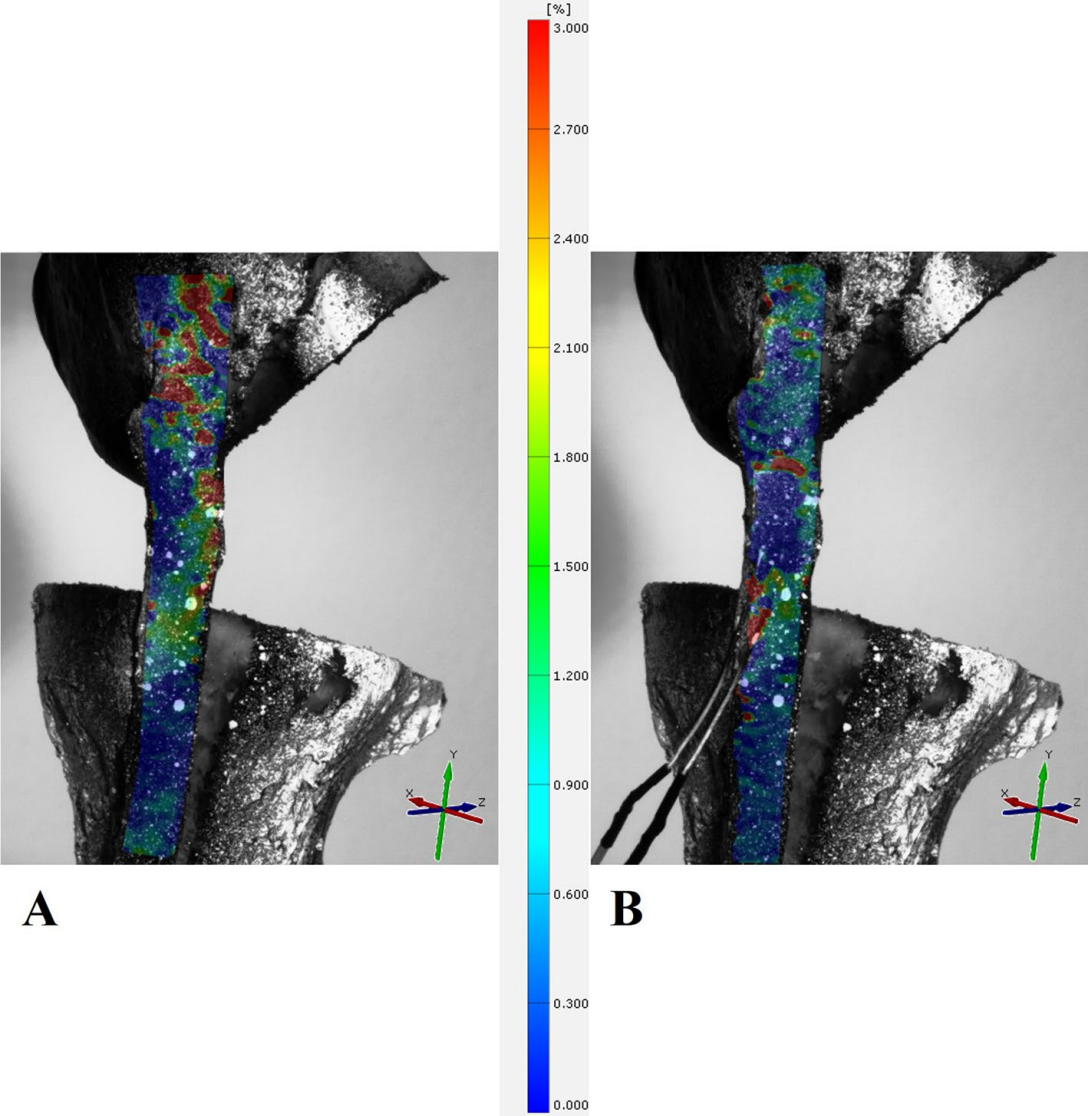
Exemplary strain map on the surface of the bone−ligament−bone complex. **A** Trial 1 and local coordinate system. **B** Trial 2 with SG directly attached to the ligament surface and local coordinate system

For the data analysis of the 3D-DIC measurement, a surface component consisting of many facets representing a measurement point was created from the whole ligament structure. The surface component of the ligaments in the second trial also included the SG based on the applied speckle pattern. A local coordinate system along the fibers was created to detect the surface strain in direction of the load applied during the test trials. Based on that, a strain map was visualized in the longitudinal direction (*e*_y_) on the surface of the ligament, showing regional strains in a color scheme over the entire test trial (Fig. 3). For subsequent analysis, the software calculated the longitudinally mean overall engineering strain during all cycles.

Additionally, the area and mean intersection deviation of the surface component was obtained during all cycles to verify the quality of the speckle pattern and the validity of the calibration.

### Statistical analysis

For all 18 specimens, the average strain of every assessment method was calculated by detecting the peak strain of each cycle. Additionally, the force of the testing machine and the area and intersection deviation of the surface component obtained with the 3D-DIC system was analyzed to represent the structural behavior of the ligaments during the tests.

The statistical analysis was performed using GraphPad Prism (9.1.2, GraphPad Software Inc., San Diego, CA, USA). The calculated data of each specimen was evaluated for normality using the Shapiro–Wilk Test. Normally distributed data were analyzed using a student’s paired *t* test. Non-normally distributed data was analyzed with a Wilcoxon Test. In addition, a comparative analysis of the correlation between the measurement techniques was performed using Bland–Altman plots**.** The difference of the bias to zero was evaluated using an one-sample *t* test [11]. For all tests, significance was set at *p* value < 0.05.

## Results

### Biomechanical evaluation of the ligaments

The mean length of the MCL measured from the center of the femoral to tibial attachment was 98.6 mm ± 8.2 mm, and the average length of the LCL measured from the center of the femoral to fibular attachment was 65.2 mm ± 6.0 mm.

**Fig. 4:**
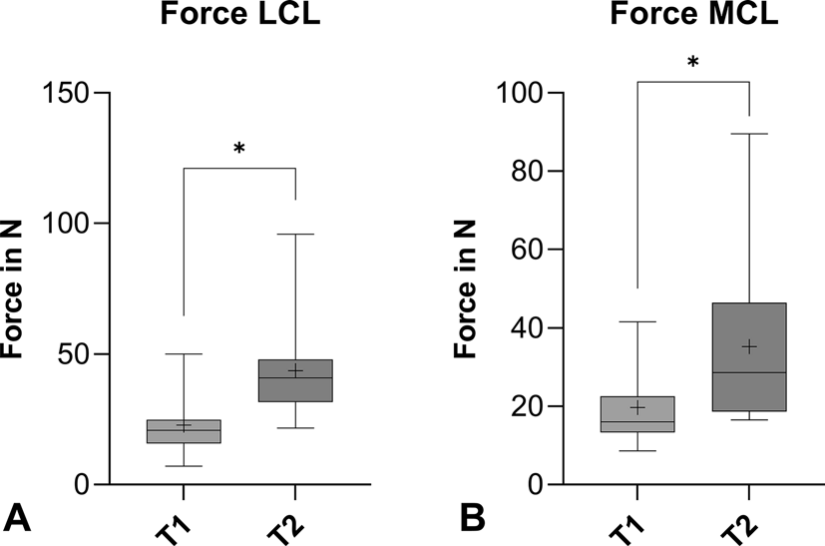
Box plots representing the force of LCL (**A**) and MCL (**B**) to reach 2 mm elongation during trial 1 (T1) and trial 2 (T2) (*p < 0.0001, + mean value)

During the first trial, the mean force to reach a 2 mm elongation of the LCL (22.8 N ± 10.5 N) was significantly (*p* < 0.0001) lower than during the second trial (43.7 N ± 20.1 N), where the SG was directly attached to the ligament surface. The behavior of the MCL was similar, with significant (*p* < 0.0001) lower forces in the first trial (19.7 N ± 10.5 N) than in the second trial (35.3 N ± 20.5 N) (Fig. 4).

### Surface component (3D-DIC) analysis

The mean area of the surface component from the LCL was in the first trial with 249.1 mm^2^ ± 96.3 mm^2^ significantly (*p* = 0.0089) higher than in the second trial with 197.8 mm^2^ ± 76.8 mm^2^. The mean area obtained from the MCL showed no significant differences from the first trial (605.5 mm^2^ ± 223.0 mm^2^) to the second trial (595.6 mm^2^ ± 209.7 mm^2^). The mean intersection deviation was 0.015 *px* ± 0.13 *px* for LCL in the first trial, 0.011 *px* ± 0.049 *px* for LCL in the second trial and 0.0062 px ± 0.069 px for MCL in the first trials as well as 0.015 px ± 0.035 px for MCL in the second trial. Overall, there was no significant difference within one trial or between trials for LCL and MCL.

### Strain analysis

**Fig. 5:**
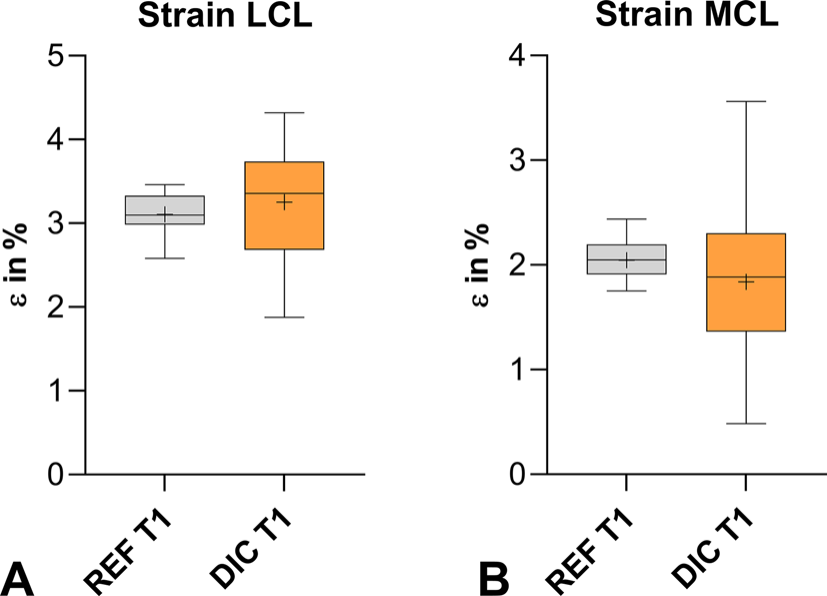
Box plot representing the strain of LCL (**A**) and MCL (**B**) measured by REF and 3D-DIC during the first trial (T1) (+mean value)

**Fig. 6:**
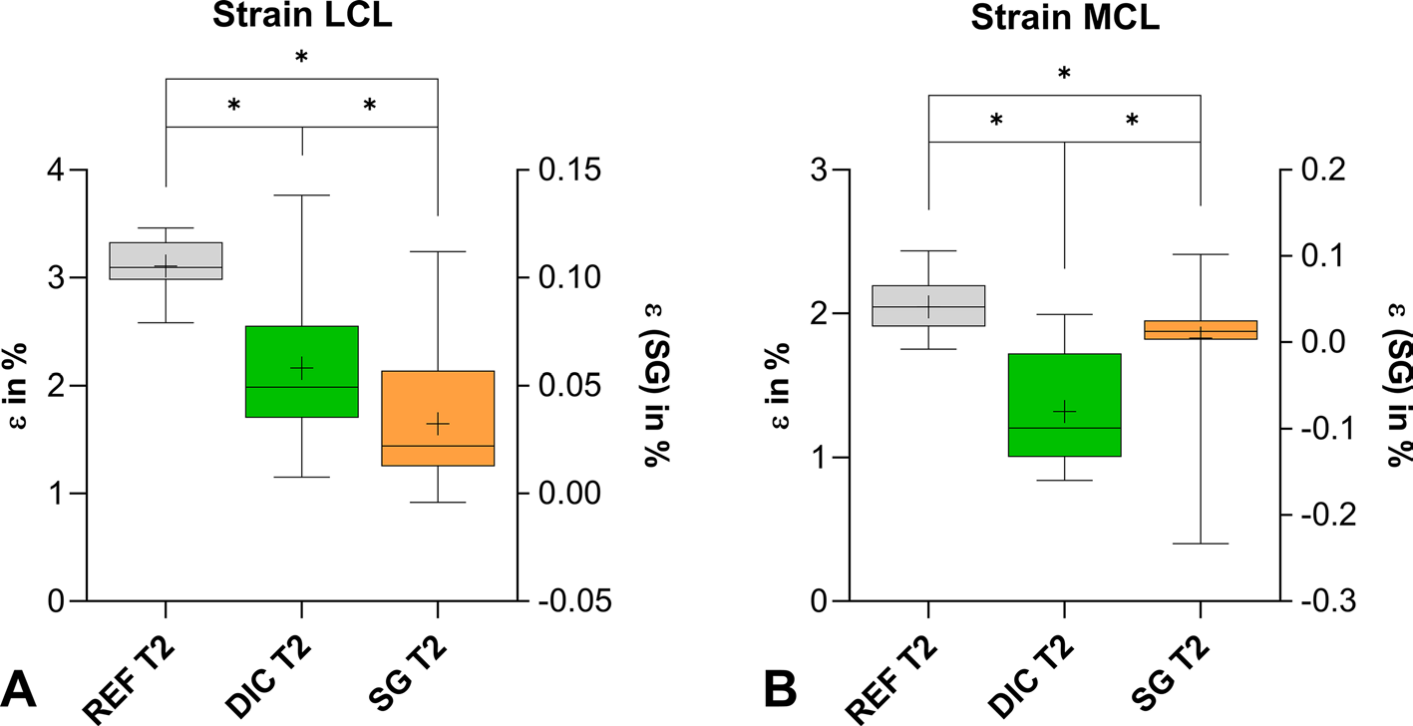
Box plot representing the strain of LCL (**A**) and MCL (**B**) measured by REF, 3D-DIC, and SG in the second trial (T2) (*p value < 0.05, second *y*-axis for SG values, + mean value)

The ɛ_REF_ measured during both test trials (T1 and T2) was not significantly different. The strains in the LCL computed by 3D-DIC were significantly (*p* = 0.0006) higher in the first trial than in the second trial. The strains in the MCL during the first trial were, similarly to the LCL, significantly (*p* = 0.0005) higher than in the second trial. Nevertheless, a comparison of the strain calculated with ɛ_REF_ and 3D-DIC showed no differences within the first trial for both LCL and MCL (Fig. 5). In the second trial, the strains assessed by SG and 3D-DIC were significantly (*p* < 0.0001) lower than those calculated by the reference. The strains measured by 3D-DIC compared to SG showed significant (*p* < 0.0001) differences (Fig. 6). Overall, the strains assessed in the LCL and MCL showed the same tendency. The individual mean values as well as the p values of the statistical analysis can be found in the Appendix Tables 2 and 3.

### Agreement analysis of the measuring systems

**Fig. 7:**
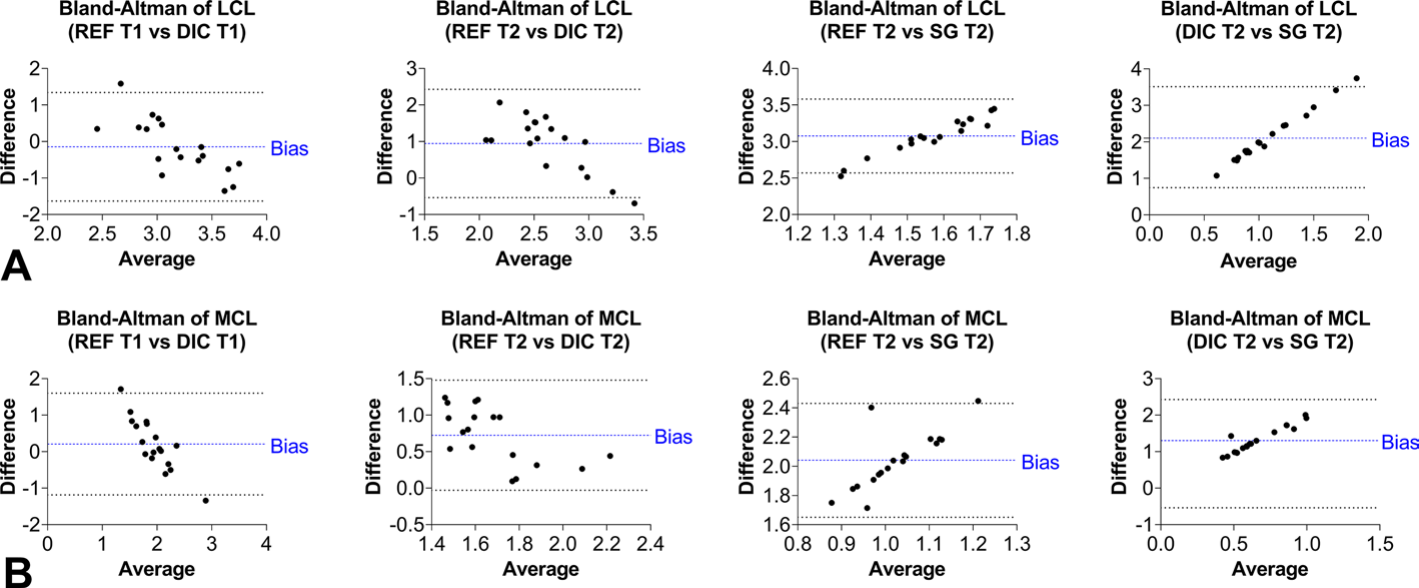
Bland–Altman plots of the different strain assessment techniques for the LCL (**A**) and MCL (**B**) with the bias (blue) and 95% limits of agreement

The Bland–Altman plots comparing the mean strains of ɛ_REF_ and 3D-DIC within the first trial had biases of 0.2% ± 0.7% for MCL and − 0.1% ± 0.8% for LCL, which were not significantly different from zero. In contrast, when comparing the mean strains of ɛ_REF_ and 3D-DIC in the second trial, the biases were 0.7% ± 0.4% for MCL and 0.9% ± 0.8% for LCL, and thus both were significantly (*p* < 0.0001) different from zero. The same significant (*p* < 0.0001) differences from zero were observed in the biases of the compared mean strains from ɛ_REF_ and SG with 2.0% ± 0.2% for MCL and 3.1% ± 0.3% for LCL. The comparison of the mean strains obtained from 3D-DIC in the second trial and SG showed biases of 2.1% ± 0.7% for LCL and 1.3% ± 0.4% for MCL, both significantly (*p* < 0.0001) different from zero (Fig. 7).

## Discussion

The most important finding of this study was that only the 3D-DIC provides results comparable to the reference. The SG measurements demonstrate significantly lower strain values in comparison to the 3D-DIC and the reference. These findings are in accordance with previous studies in strain assessment using 3D-DIC method on ligaments [22, 24, 31, 36]. On the other hand, the values measured with SGs showed a slightly lower strain in the MCL compared to the LCL. which results in a possible qualitative strain analysis for obtaining differences between various experiments. Furthermore, SGs can be used for repeatable measurements within the same specimen, which is in accordance with other studies about strain assessment using foil-type SG on ligaments [4, 28].

The 3D-DIC method to measure strain on human soft tissue is a valid method when a highly contrasted speckle pattern, an unrestricted view, and a proper alignment of the ligament within the setup are achieved [22, 24, 31, 36]. A common way to verify these parameters is the measurement of the mean intersection deviation. In this study, the mean intersection deviation was lower or equal to 0.01 pixels across specimen, whereas the strain could be recorded with the same appropriate resolution. During testing, the effect of delamination and crumbling of the paint layer due to small deformations was not visible, and the test was performed immediately before the paint dried completely. In addition, tissue alterations were minimized with the use of water-based paints [10]. Nevertheless, the comparison between 3D-DIC and the reference showed a slightly difference due to the superficial measurement of 3D-DIC compared to the reference, which included the strain of the whole ligamental structure. The surface detected by the 3D-DIC software was also influenced by the applied speckle pattern. The surface component visualizes the strain distribution of the superficial ligament during the tests, with the highest strain values near the insertion sites [12]. By applying the SG to the surface of the ligament, the perceptibility is affected by the cables and the SG itself, which causes a significantly underestimation of the strain measured by 3D-DIC This effect was more prominent in the thin LCL than in the larger MCL. Moreover, the force required for 2 mm elongation increased after the application of the SG. Considering these changes and a varying strain distribution observed by the 3D-DIC, the ligaments might have been stiffened due to glue penetration and tissue infiltration.

However, once applied, the circuit of the SG is in tension, which can be minimized by calibrating the bridge circuit. Shunt calibration can be used to minimize wiring faults or damaged wires, enabling offset zero balance of the bridge to 0 V output when no strain is present [15]. In the past, the use of SGs has not been promising due to the restoring forces in the SG and the difficulty of accurate displacement calibration without knowing the correct strain characteristics of each measurement point [15, 35]. In general, the proper alignment of the SG parallel to the direction of the fibers is essential to minimize this influence. Furthermore, as stress in the transverse direction also causes resistance changes and affects the resulting strain, measurements using single foil-type SG can only be performed longitudinally to produce reproducible results [4, 25]. Besides the previous mentioned challenges which might have had an influence on the variability in strain estimation between the methods, SGs measure strain only in a small region of interest and within a different imaging plane compared to 3D-DIC.

One of the downsides of this study is the order of our experimental tests, which may result in systematic errors. Based on the absolute elongation of the testing machine, the length of the LCL increased by approximately 0.8 mm ± 0.4 mm and the length of the MCL increased by approximately 0.9 mm ± 0.3 mm from the beginning of the first trial to the beginning of the second trial. In addition, the force between the trials was also increased due to the testing protocol. By taking these length differences into account when calculating the reference strain, the mean strain is equivalent to the previously calculated one. Therefore, errors are not considered when comparing the 3D-DIC and SG method with the reference. In addition, the setup tested displacement control up to 2 mm to ensure comparability. Another downside of this study is the simple ex vivo loading scenario up to an elongation of 2 mm with an elongation rate of 20 mm/min. Combined with 3D-DIC and SG as strain measurement methods that only analyze the superficial layer of tissue, it results in difficulties in clinical translation as well as further challenges in data collection due to complex in vivo loading conditions and anatomy. Nevertheless, the elongation of 2 mm resulted in a percentage strain of about 2% in the MCL and 3% in the LCL. Those strains are within the elastic behavior of ligaments, hence, the influence on the ligaments due to the testing parameters were minimized.

Moreover, the effect of other components of the setup on the strain measurement were not considered because of the differences in stiffnesses between the bony parts (900–1500 N/mm), test rig (4653.1 N/mm ± 52.7 N/mm) and ligaments (30–90 N/mm) [3, 13, 39, 41]. Another limitation of the present study is the reuse of the specimens, with at least three freeze–thaw cycles. However, referring to Woo et al. and Moon et al. re-freezing has no influence on the mechanical behavior of the tissue [26, 45].

## Conclusion

According to our results, both systems allow strain assessment within the range of elastic behavior of the ligament, however only the 3D-DIC provides results comparable to the reference. As described in literature, the 3D-DIC method provides quantitative and qualitative results through full-field analysis of superficial ligaments. It also provides an overview of the regional strain distribution. On the other hand, the strains measured by directly attached SGs are not comparable to the 3D-DIC system. Nevertheless, SGs can be used to detect local longitudinal strain differences. Due to the simple fixation method using superglue, SGs can be used to study the behavior of ligaments in joints, even if they are not superficial, and especially if they are optically inaccessible for 3D-DIC.

## Data Availability

The datasets used and analyzed during the current study are available from the corresponding author on reasonable request.

## Appendix

The mean strain and standard deviation of LCL and MCL calculated by the reference, 3D-DIC and SG are shown in Table 2. Strain data from the testing machine (REF) and the 3D-DIC system were normally distributed and analyzed using a Student’s paired *t* test, and the non-normally distributed SG data were analyzed using a Wilcoxon’s test (Table 3).

**Table 2:** Mean strain and standard deviation of the LCL and MCL depending on the assessment method

	LCL	MCL
REF—Trial 1 (%)	3.1 ± 0.3	2.1 ± 0.2
REF—Trial 2 (%)	3.1 ± 0.3	2.1 ± 0.2
3D-DIC—Trial 1 (%)	3.3 ± 0.7	1.8 ± 0.7
3D-DIC—Trial 2 (%)	2.2 ± 0.7	1.3 ± 0.4
SG—Trial 2 (%)	0.03 ± 0.03	0.005 ± 0.06

**Table 3:** P values of LCL and MCL from the statistical analysis

Analysis parameters	*p* value (LCL)	*p *value (MCL)
REF T1—REF T2	0.44	0.11
REF T1—3D-DIC T1	0.43	0.23
REF T2—3D-DIC T2	< 0.0001*	< 0.0001*
REF T2—SG T2	< 0.0001*	< 0.0001*
3D-DIC T2—SG T2	< 0.0001*	< 0.0001*

*Significant difference
